# PPP2R1B abolishes colorectal cancer liver metastasis and sensitizes Oxaliplatin by inhibiting MAPK/ERK signaling pathway

**DOI:** 10.1186/s12935-024-03273-w

**Published:** 2024-03-01

**Authors:** Wei Liu, Jingtong Tang, Wei Gao, Jian Sun, Gang Liu, Jianping Zhou

**Affiliations:** 1 Department of Gastrointestinal Surgery, The First Hospital of China Medical University, Nanjing Street 155, Shenyang, 110001 China; 2 Department of General Surgery, Daqing Oilfield General Hospital, Daqing, 163000 China; 3 Shenyang Medical Nutrition Clinical Medical Research Center, Shenyang, China

**Keywords:** PPP2R1B, Colorectal cancer, Liver metastasis, Oxaliplatin, p-ERK

## Abstract

**Background:**

Patients with colorectal cancer (CRC) with liver metastasis or drug resistance have a poor prognosis. Previous research has demonstrated that PPP2R1B inactivation results in the development of CRC. However, the role of PPP2R1B in CRC metastasis and drug resistance is unclear.

**Methods:**

Venny 2.1 was used to determine the intersection between survival-related differentially expressed genes (DEGs) and liver metastasis-related DEGs according to RNA-seq data from The Cancer Genome Atlas (TCGA) and the GEO database (GSE179979). LC‒MS/MS and coimmunoprecipitation were performed to predict and verify the substrate protein of PPP2R1B. Gene Set Variation Analysis (GSVA) was subsequently utilized to assess pathway enrichment levels. The predictive performance of PPP2R1B was assessed by regression analysis, Kaplan–Meier (KM) survival analysis and drug sensitivity analysis. Immunohistochemistry (IHC), qRT-PCR and western blotting were performed to measure the expression levels of related mRNAs or proteins. Biological features were evaluated by wound healing, cell migration and invasion assays and CCK-8 assays. A mouse spleen infection liver metastasis model was generated to confirm the role of PPP2R1B in the progression of liver metastasis in vivo.

**Results:**

According to bioinformatics analysis, PPP2R1B is significantly associated with liver metastasis and survival in CRC patients, and these findings were further verified via immunohistochemistry (IHC), qPCR and Western blotting. Pathway enrichment and LC‒MS/MS analysis revealed that PPP2R1B is negatively associated with the MAPK/ERK signalling pathway. Additionally, PD98059, a MAPK/ERK pathway inhibitor, inhibited EMT in vitro by reversing the changes in key proteins involved in EMT signalling (ZEB1, E-cadherin and Snail) and ERK/MAPK signalling (p-ERK) mediated by PPP2R1B. Oxaliplatin sensitivity prediction and validation revealed that PPP2R1B silencing decreased Oxaliplatin chemosensitivity, and these effects were reversed by PD98059 treatment. Moreover, PPP2R1B was coimmunoprecipitated with p-ERK in vitro. A negative correlation between PPP2R1B and p-ERK expression was also observed in clinical CRC samples, and the low PPP2R1B/high p-ERK coexpression pattern indicated a poor prognosis in CRC patients. In vivo, PPP2R1B silencing significantly promoted liver metastasis.

**Conclusions:**

This study revealed that PPP2R1B induces dephosphorylation of the p-ERK protein, inhibits liver metastasis and increases Oxaliplatin sensitivity in CRC patients and could be a potential candidate for therapeutic application in CRC.

**Supplementary Information:**

The online version contains supplementary material available at 10.1186/s12935-024-03273-w.

## Introduction

Colorectal cancer (CRC) is one of the leading causes of cancer-related morbidity and mortality worldwide, and its pathogenesis involves a myriad of genetic and epigenetic alterations [1]. Approximately 25–30% of CRC patients are diagnosed with CRC and liver metastasis at the same time or asynchronously [2]. Although surgery or adjuvant chemotherapy is often used for treatment, CRC patients with liver metastasis have poor survival [3]. Moreover, both drug resistance and metastasis seriously affect the prognosis of CRC [4]. Hence, the mechanisms of CRC metastasis and drug resistance need to be investigated thoroughly.

Phosphatase 2A (PP2A), a predominant cellular serine-threonine phosphatase, is reported to play a critical role in fundamental cancer cellular processes [5]. Recent studies have demonstrated that the PPP2R1B gene, which encodes PP2A subunit A, is altered in human lung and colorectal carcinomas [6]. Moreover, Dinoi G and Cui J reported that PPP2R1B is related to cancer cell metastasis and drug resistance [7, 8]. However, the mechanism of action of PPP2R1B in cancer progression is poorly understood, and the target substrate of PPP2R1B has not been verified.

Epithelial-to-mesenchymal transition (EMT) is a dynamic and complicated process. Moreover, EMT has been implicated in cancer metastasis and drug resistance [9, 10]. Numerous signalling pathways have been demonstrated to play pivotal roles in the progression of EMT, and the mitogen-activated protein kinase (MAPK) pathway—especially its downstream effector, phosphorylated extracellular signal-regulated d kinase (p-ERK)—is a key pathway because of its central role in modulating cellular processes crucial for tumour growth and progression [11, 12]. Hence, inhibiting MAPK/ERK signalling has become a focus of cancer research [13].

In this study, we found that a novel PPP2R1B/MAPK/ERK signalling axis mediates CRC cell metastasis and drug resistance and that p-ERK may be a target of PPP2R1B. This study aimed to comprehensively characterize PPP2R1B in the context of CRC liver metastasis, covering its molecular dynamics, clinical implications, and therapeutic target potential. Through a detailed exploration of the abovementioned molecular pathway, we aimed to highlight promising strategies for suppressing CRC progression and improving patient prognosis.

## Materials and methods

### Human tissue specimens and cell lines

We obtained 100 pairs of fresh CRC tissues and corresponding adjacent nontumor colorectal tissues from patients (57 men and 43 women) with a median age of 64.7 years (range, 41 to 80 years). These samples were collected after CRC surgery from the Department of Gastrointestinal Surgery, the First Hospital of China Medical University. All specimens were pathologically confirmed to be CRC, and the data were categorized according to the 8th UICC guidelines. Informed consent was acquired from some patients, while others were exempted from providing informed consent given the assurance of anonymity. This procedure was approved by the ethics committee of the First Hospital of China Medical University (2023[95]). This study adhered to the guidelines set by the Declaration of Helsinki.

The mRNA expression data, excluding the expression of PPP2R1B in the extremities, and clinicopathological annotations of 362 CRC patients were extracted from The Cancer Genome Atlas (TCGA) database (http://cancergenome.nih.gov), which includes information on tumour samples and paracancerous samples with detailed information for further analysis. Additionally, data for 4 primary tumour and 3 liver metastasis samples from CRC patients (GSE179979) with detailed characteristic information were obtained from the GEO database (https://www.ncbi.nlm.nih.gov/geo/).

Five human CRC cell lines (HCT116, SW480, HCT8, RKO and LOVO) were procured from a cell bank at the Chinese Academy of Sciences (Shanghai, China), one normal colon cell line (NCM460) was acquired from the BeNa Culture Collection (Beijing, China), and one murine MC38 cell line was purchased from the National Infrastructure of Cell Line Resource (Beijing, China). The cells were cultured in the recommended growth media supplemented with 10% foetal bovine serum (FBS; HyClone, Logan, UT, USA) and 100 U/m penicillin or streptomycin. These cells were maintained in a humidified chamber containing 5% CO2 at 37 °C. PD98059 (2-(2-amino-3-methoxyphenyl)-4H-1-benzopyran4-one), a non-ATP competitive selective MEK1/2 inhibitor that specifically inhibits MEK-1-mediated activation of MAPK, was purchased from Sigma Chemical Co. (St. Louis, USA), and Oxaliplatin was purchased from Selleck (Shanghai, China). The cells were treated with PD98059 (10 µM for 24 h) or Oxaliplatin (10 µM for 24 h).

### RNA extraction and quantitative real-time PCR

Total RNA was extracted from fresh CRC cell lines and colorectal tissue preserved in liquid nitrogen using TRIzol (Takara, Japan) according to the manufacturer’s instructions. The concentration and purity of the RNA were determined using a NanoDrop 1000 (Thermo Scientific, USA). The expression levels of both PPP2R1B and GAPDH were measured using QuantStudio 3 (Applied Biosystems, USA). Reverse transcription and amplification were performed using a kit from Takara. The thermocycling conditions were set to 95 °C for 30 s, followed by 45 cycles of 95 °C for 5 s and 60 °C for 34 s.

### Protein isolation and Western blotting

Total protein was extracted from CRC cell lines and tissues using RIPA lysis buffer supplemented with 1% PSMF and 1% protease inhibitor. The protein samples were separated on 10% sodium dodecyl sulfate‒polyacrylamide gels and subsequently transferred to PVDF membranes. These membranes were blocked with 5% nonfat milk for 2 h, followed by incubation with the following antibodies: rabbit PPP2R1B at a dilution of 1:1000 (Proteintech Group, China), rabbit p-ERK at 1:1000 (Abmart, China), rabbit ERK at 1:1000 (PTMBIO, China), rabbit E-cadherin at 1:1000 (Proteintech Group, China), rabbit ZEB1 at 1:1000 (Proteintech Group, China), rabbit Snail at 1:500 (Proteintech Group, China), and mouse GAPDH at 1:3000 (Proteintech Group, China). The following day, the membranes were incubated with their corresponding secondary antibodies at 1:10,000 (Proteintech Group, China) for 2 h. Each experiment was conducted in triplicate.

### HE staining

After deparaffinization and rehydration, 4 µm sections were stained with haematoxylin solution for 6 min, followed by 8 s in 1% acid ethanol (1% HCl in 70% ethanol) and rinsing in distilled water. The sections were subsequently stained with eosin solution for 3 min, dehydrated with graded alcohol and cleared in xylene. Finally, images were captured using a microscope (Nikon, Japan).

### Immunohistochemistry (IHC)

The expression of PPP2R1B and p-ERK was assessed using immunohistochemistry (IHC). 4 µm sections were processed according to the protocol outlined by the IHC Biotin Block Kit (MXB Biotechnologies, Fuzhou, China), as described by Tang et al. [14]. Initial evaluations of staining were conducted at low magnification (×10) and subsequently confirmed at higher magnifications (×20). The expression of the proteins was scored by both the percentage of positive cells and staining intensity. Staining heterogeneity was graded as follows: 0 (≤ 5%), 1 (6–25%), 2 (26–50%), or 3 (> 51%). Based on these scores, PPP2R1B and p-ERK expression levels were computed. Two independent pathologists critically evaluated the final scores.

### Coimmunoprecipitation

Coimmunoprecipitation was performed as described in a previous study [15]. The lysis buffer was composed of 20 mM Tris/HCl (pH 7.4), 1.0% NP40, 150 mM NaCl, 1 mM EDTA, 10 μg/mL leupeptin, and 50 μg/mL PMSF. Using this lysis buffer, we extracted protein from SW480 cells (treated with or without Oxaliplatin). Antibody beads were preincubated with magnetic beads (Bio-Rad, Hercules, CA, USA). Additionally, 200 μl of rabbit PPP2R1B antibody (Proteintech Group, China) or IgG (Santa Cruz, Japan) at a 1:100 dilution was incubated with the SW480 total protein at 4 °C overnight. Western blotting was then performed to visualize the immunoprecipitate samples separated using magnetic beads the next day.

### siRNA and plasmid synthesis and transfection

The siRNAs targeting PPP2R1B, as well as the negative control (NC), were obtained from GenePharma (Shanghai, China). The specific sequences used were as follows: The PPP2R1B plasmid was synthesized by Genechem (Shanghai, China). HCT116 and SW480 CRC cells were transfected with PPP2R1B si1, PPP2R1B si2, negative control (NC-siRNA) or the PPP2R1B plasmid using jetPRIME reagent (Polyplus, France) in accordance with the provided guidelines. After 48–72 h of incubation, the effects of siRNA transfection or overexpression were assessed via Western blotting.

### Invasion and migration assays

In brief, transfected HCT116 and SW480 cells were seeded onto membrane inserts with 8.0 μM pore size in 24-well plates filled with FBS-free growth media. Growth media containing 10% FBS served as a chemoattractant in the bottom wells. After 24 h, nonmigrated cells on the top side of the inserts were removed using a cotton swab. Cells that migrated to the underside of the inserts were stained with crystal violet hydrate (Solarbio, China) following the manufacturer’s guidelines. An invasion assay was similarly conducted, and the cells were evaluated using modified Boyden chamber assays (BD Biosciences, USA). Migrated cells were counted under a microscope at × 20 magnification, and images were captured using a microscope (Nikon, Japan). Cells were quantified in five random fields per insert, and the results are presented as the number of migrated cells per field.

### Wound healing assay

Transfected HCT116 and SW480 cells were seeded in 6-well plates. Once the cells reached 80% confluence, the bottom of the plate was gently scraped using a 200 µl pipette tip. The plate was then washed with PBS three times, and images were captured at 0 and 24 h using a microscope (Nikon, Japan) at a 10 × magnification.

### Pathway analysis

Pathway scores were calculated using the gene set variation method (GSVA) followed by ordinal regression [16]. The GSVA score was generated according to the mRNA expression level. To identify PPP2R1B-related pathways, we used the pathway score generated by GSVA in conjunction with the PPP2R1B expression level in the same dataset.

### Drug response prediction and detection

We mainly used a ridge regression-based method of the R package oncoPredict (version 0.2) to predict the IC50 AUC values of potential drug responses which bridges the in vitro drug screening with in vivo drug and biomarker discovery [17]. The Oxaliplatin sensitivity of the transfected CRC cells was detected using CCK-8 assays. First, cells that were seeded in 96-well plates (Servicebio, Wuhan, China) were incubated with 10 µM Oxaliplatin (Selleck, China) for 24 h. Next, 10 µl of CCK-8 reagent (Dojindo, Kumamoto, Japan) was added, and the cells were incubated at 37 °C for 2 h. Finally, the absorbance was measured at 450 nm in an ELISA 96-well microtiter plate reader (Bio-Rad 680, California, USA).

### Shotgun LC‒MS/MS

Shotgun LC‒MS/MS was performed by Genechem (Shanghai, China). The protein solution was digested by protease, after which the peptides were obtained. The peptides were then separated using high-performance liquid chromatography (HPLC) and added to a high-resolution mass spectrometer for analysis. The use of retrieval software in conjunction with the corresponding proteome database for the analysis of mass spectrum data enables the visualization of proteome information from the sample.

### Mouse xenograft model

Experiments involving mice were approved by the Ethics Committee of China Medical University. For the liver metastasis model, 6-week-old C57BL/6 mice were purchased from Liaoning Changsheng Biotechnology (Benxi, China). A small incision was made in the left abdominal flank to expose the spleen. MC38 cells transfected with NC-siRNA or PPP2R1B-siRNA1 (1 × 10^6^) suspended in prechilled PBS (100 µl) were injected into the inferior pole of the spleen. Twenty-one days later, the mice were euthanized, and liver tissues were harvested. Tumours were measured using Vernier callipers. Subsequently, liver metastatic tumours were identified using haematoxylin and eosin (HE) staining and detected via Western blotting.

### Statistical analysis

Continuous data are presented as the mean ± SE. For two groups, Student’s t test was used to evaluate differences in qRT‒PCR, WB, cell migration and invasion, wound healing and liver metastasis. Nonparametric and Spearman correlation tests were used for IHC analysis of human CRC samples. The associations of target protein expression with clinicopathological data were analysed by the chi-square test or Fisher's exact test. The Kaplan‒Meier curve was used to estimate survival, and differences were analysed by the log-rank test. R language v4.2.2 (https://www.r-project.org/) and GraphPad Prism 5.0 software were used for data analysis. P values were adjusted for multiple comparisons when necessary. P value < 0.05 was considered to indicate statistical significance.

## Results

### Ectopic expression of PPP2R1B mRNA and protein in CRC tissue, CRC liver metastasis tissue and CRC cells

**Fig. 1:**
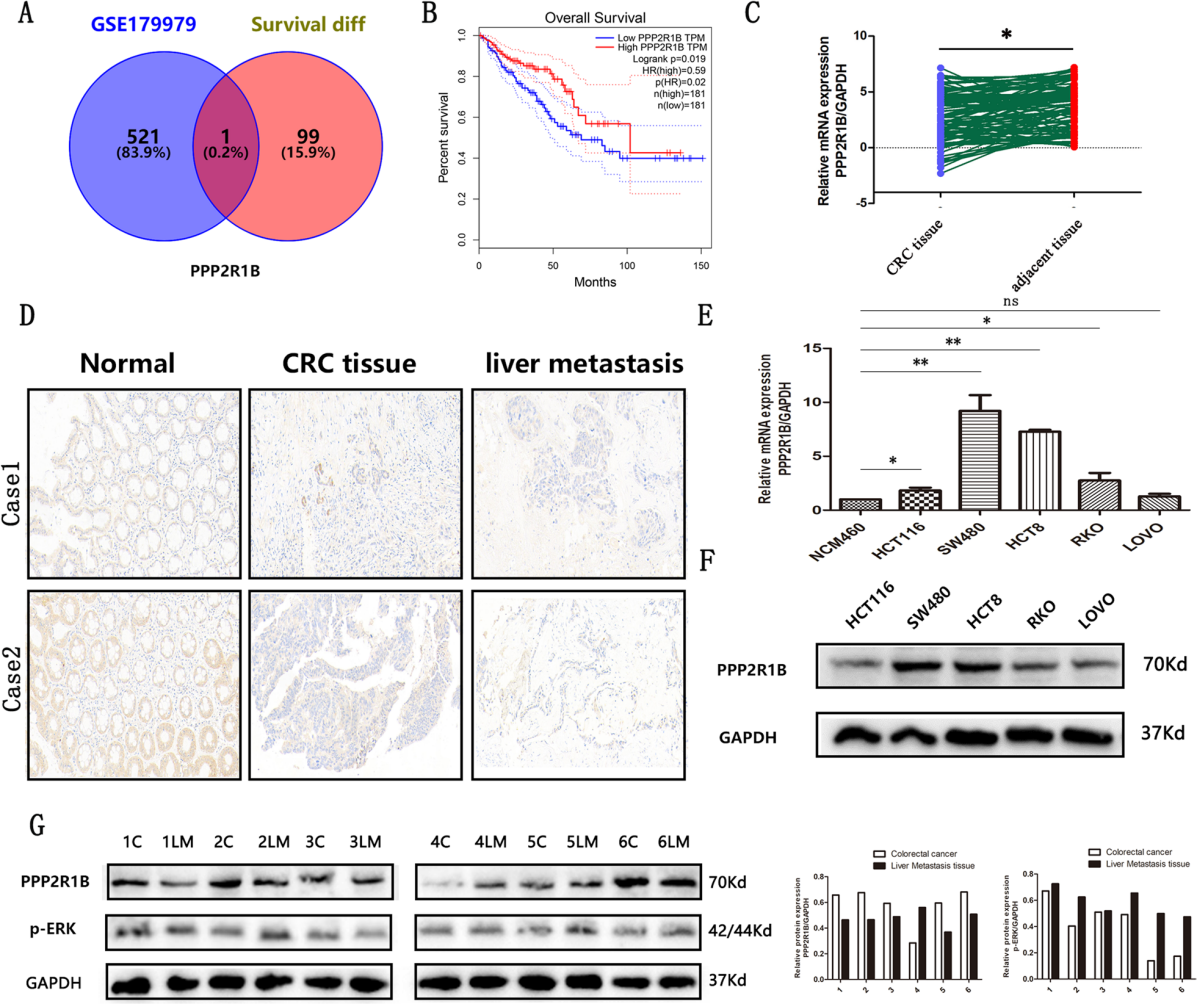
PPP2R1B expression in CRC cells, CRC tissues and liver metastasis tissues. **A** The intersection genes between differential genes in GSE179979 dataset and 100 survival-related genes in TCGA database by Venny diagram. **B** High and low PPP2R1B expression plotted against overall survival time in 362 CRC patients in TCGA database. **C** mRNA expression of PPP2R1B in 100 pairs of CRC tissues and adjacent tissues by qRT-PCR. **D** Protein expression of PPP2R1B in CRC tissues, CRC liver metastasis and adjacent tissues by IHC. © mRNA expression of PPP2R1B in 5 CRC cell lines and 1 normal intestinal epithelial cells by qRT-PCR. **F** Protein expression of PPP2R1B in 5 CRC cell lines by WB. **G** Protein expression of PPP2R1B and p-ERK in 6 pairs of CRC and liver metastasis by WB. Bars indicate Mean ± SE, *P < 0.05, **P < 0.01 and ***P < 0.001 compared with the control

Initially, we found that PPP2R1B is the only gene that is both ectopically expressed in liver metastasis tissues (GSE179979) and related to patient survival in the TCGA cohort (Venn diagram, Fig. 1A), which suggests that PPP2R1B may play an important role in the metastasis of CRC. Hence, we selected PPP2R1B for further investigation; we also found that it was negatively associated with survival in the GDC TCGA colon cancer (COAD) cohort (Fig. 1B). To validate the bioinformatic findings, we utilized qRT‒PCR and IHC to evaluate PPP2R1B expression in colorectal adjacent tissues, CRC tissues and liver metastasis tissues. Our findings revealed that PPP2R1B is expressed at lower levels in cancerous tissues and is expressed at even lower levels in liver metastasis tissues at the mRNA and protein levels (Fig. 1C, D). As presented in Table 1, there was a negative correlation between PPP2R1B expression and both TNM stage and distant metastasis (Table 1, p = 0.02, p = 0.03). To further investigate the biological function of PPP2R1B through in vitro experiments, we evaluated its expression in five CRC cell lines and one normal intestinal epithelial cell line. Notably, qRT‒PCR and Western blotting revealed that PPP2R1B was highly expressed in SW480 cells but was expressed at low levels in HCT116 cells (Fig. 1E, F). Based on these findings, we selected the SW480 and HCT116 cell lines for subsequent experiments. Finally, we examined PPP2R1B and p-ERK protein expression in 6 pairs of CRC tissues and liver metastasis tissues, and the Western blotting results showed that PPP2R1B was expressed at lower levels in liver metastasis tissues than in CRC tissues and was negatively associated with p-ERK (Fig. 1G).

**Table 1:** The relationship between PPP2R1B and 100 CRC patients clinical pathology data

Parameters	No. of patients	Expression of PPP2R1B		P value
		Low (62)	High (38)	
Age (years)				0.82
< 60	42	25	17	
≥ 60	58	37	21	
Gender				0.73
Male	57	34	23	
Female	43	28	15	
Size (maximal diameter)				0.68
≥ 5 cm	46	30	16	
< 5 cm	54	32	22	
Differentiation				0.07
Well, moderate	60	42	18	
Poor	40	20	20	
TNM stage				0.02
I + II	58	30	28	
III + IV	42	32	10	
Tumor invasion				0.22
T1 + T2 + T3	69	46	23	
T4	31	16	15	
Lymph node status				0.33
Positive	60	40	20	
Negative	40	22	18	
Metastasis				0.03
M0	83	47	36	
M1	17	15	2	

### Manipulating PPP2R1B expression regulates CRC cell migration and invasion

**Fig. 2:**
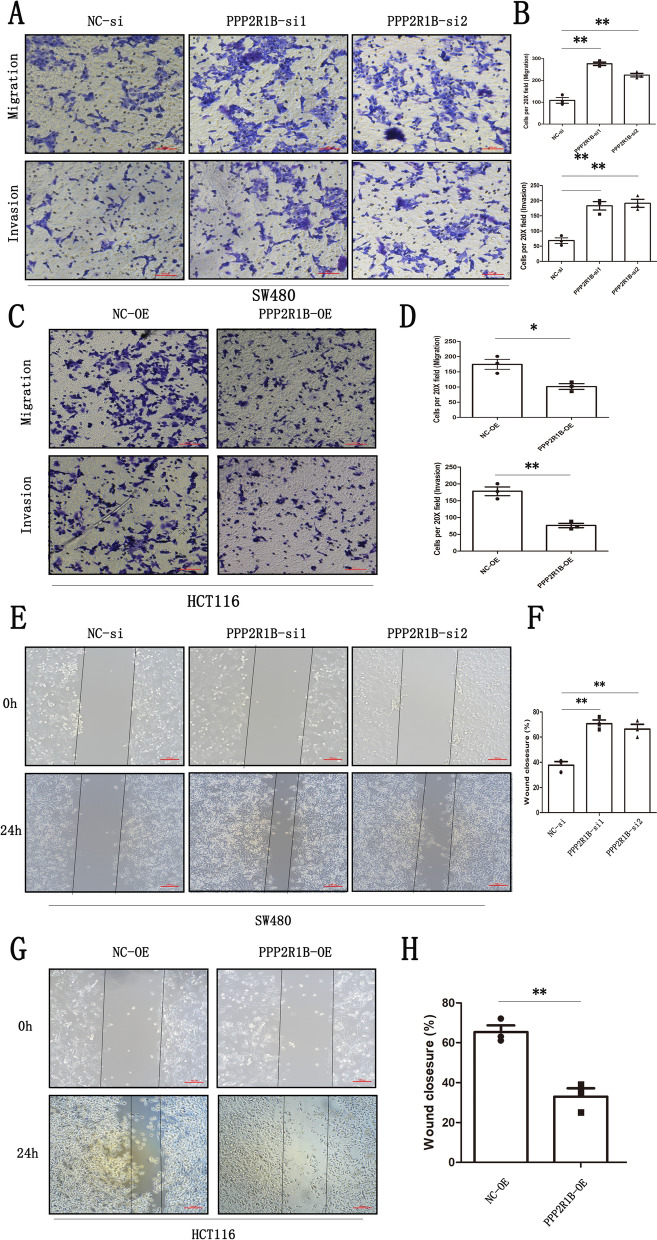
PPP2R1B inhibited CRC cell invasion and migration. **A** Cell migration and invasion assay in SW480 transfected NC siRNA or PPP2R1B siRNA. **B** Cell migration and invasion assay in HCT116 transfected PPP2R1B plasmid. **C** Wound healing assay in SW480 transfected NC siRNA or PPP2R1B siRNA. **D** Wound healing assay in HCT116 transfected PPP2R1B plasmid. The cells were showed under a microscope at ×20 magnification in migration and invasion assay, and at ×10 magnification in Wound healing assay. Bars indicate Mean ± SE, *P < 0.05, **p < 0.01 and ***P < 0.001 compared with the control, n = 3

CRC metastasis results in poor survival in CRC patients [3]. We performed invasion, migration, and wound healing assays to verify the effects of PPP2R1B on CRC cell invasion and migration in two CRC cell lines. The number of migrated cells and the changes in gap distance in the wound healing assay indicate CRC cell invasion and migration ability. The results showed that silencing PPP2R1B promoted CRC cell invasion and migration in SW480 cells (Fig. 2A, B). Moreover, overexpression of PPP2R1B impaired CRC cell invasion and migration in HCT116 cells (Fig. 2C, D).

### PPP2R1B silencing increases EMT-related marker expression and stabilizes p-ERK in CRC

**Fig. 3:**
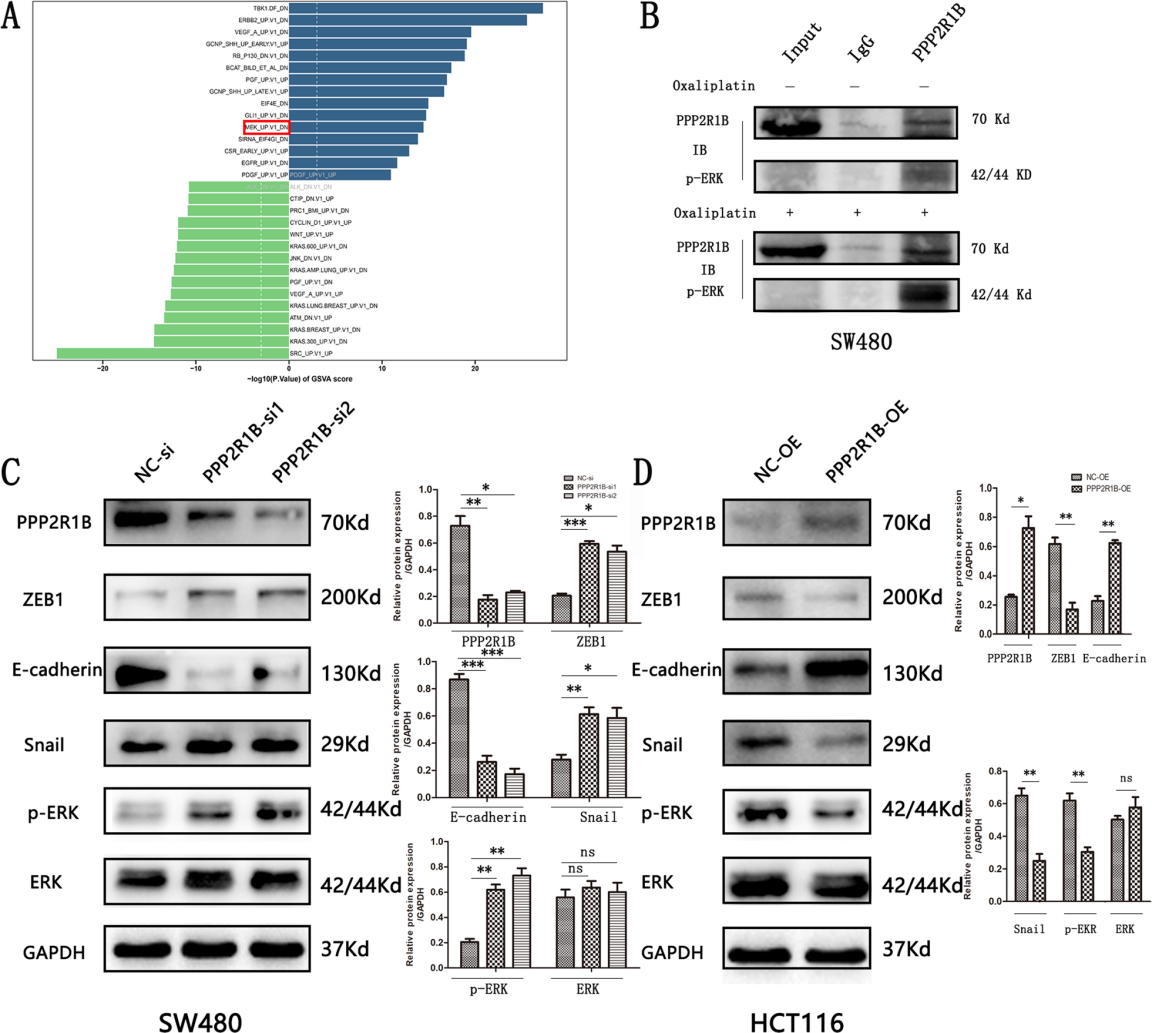
PPP2R1B induced dephosphorylation of p-ERK and metastasis related markers. **A** The GSVA results showed that PPP2R1B is negatively associated with MEK signaling pathways. **B** p-ERK co-immunoprecipitated with PPP2R1B in SW480 CRC cells with or without Oxaliplatin. **C** protein expression involving PPP2R1B, ZEB1, E-cadherin, p-ERK, ERK, Snail in PPP2R1B siRNA group and negative control group in SW480. **D** The protein expression involving PPP2R1B, ZEB1, E-cadherin, p-ERK, ERK, Snail in PPP2R1B overexpression plasmid group and negative control group in HCT116. Bars indicate Mean ± S.E. *P < 0.05, **P < 0.01 and ***P < 0.001 compared with the control, n = 3

The mechanism by which PPP2R1B inhibits CRC metastasis was explored. First, through GSVA analysis, we found that PPP2R1B is negatively related to the MEK signalling pathway, which indicates that PPP2R1B expression decreases with the enrichment of this pathway (Fig. 3A). Moreover, mass spectrometry indicated that the PPP2R1B protein binds to the ERK protein (Additional file 1: Table S1). PPP2R1B may act as an inhibitor of the MAPK/ERK signalling pathway, which is associated with EMT and plays an important role in the migration and invasion of CRC cells and in the distant metastasis of CRC [18].

Subsequently, protein immunoprecipitation (co-IP) assays were performed on SW480 cells treated with or without Oxaliplatin. The results showed that PPP2R1B could bind to p-ERK in SW480 cells. Moreover, Oxaliplatin promoted the interaction between these two proteins (Fig. 3B).

Finally, we detected ERK, p-ERK, E-cadherin and other EMT-related markers by Western blotting. According to the PPP2R1B expression pattern in CRC cell lines, we inhibited PPP2R1B expression in SW480 cells and increased PPP2R1B expression in HCT116 cells. We found that the expression of p-ERK, ZEB1 and Snail increased and that the expression of E-cadherin decreased when PPP2R1B was silenced in SW480 cells, while the expression of ERK did not change (Fig. 3C). Moreover, compared with PPP2R1B silencing, PPP2R1B overexpression had the opposite effect on p-ERK and the protein expression of ZEB1, Snail and E-cadherin in HCT116 cells (Fig. 3D).

### PPP2R1B inhibits CRC cell invasion and migration via the MAPK/ERK signalling pathway

**Fig. 4:**
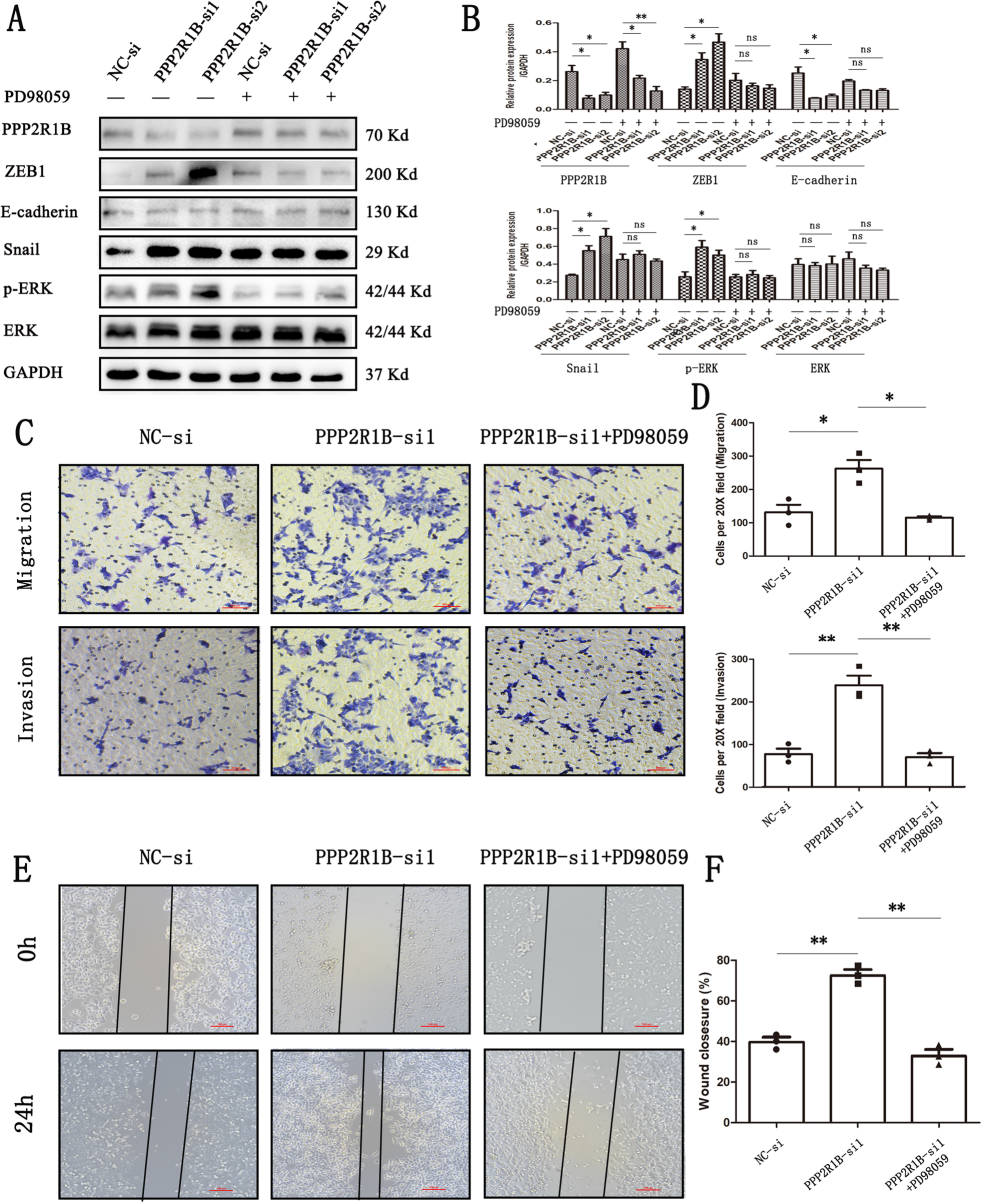
PPP2R1B regulates CRC cells metastasis by MAPK/ERK signaling pathway. **A** Protein of PPP2R1B, ZEB1, E-cadherin, Snail, p-ERK and ERK change in SW480 in PPP2R1B siRNA group and negative control group with or without PD98059. **B** Quantitative analysis of the Western Blot was performed for SW480 cells using ImageJ software. **C** Representative images of SW480 cells transfected with control, PPP2R1B knockdown and PPP2R1B knockdown plus PD98059 in migration and invasion assays at ×20 magnification. **D** Quantitative analysis of the numbers of cells was performed for SW480 cells using ImageJ software. **E** A wound healing assay was performed in SW480 cells with control, PPP2R1B knockdown and PPP2R1B knockdown plus PD98059. Representative images at the indicated time points are shown at magnification ×10. **F** Quantitative analysis of the migration area was performed for SW480 cells using ImageJ software. Bars indicate Mean ± S.E. *P < 0.05, **P < 0.01 and ***P < 0.001 compared with the control, n = 3

To further assess whether PPP2R1B could regulate the MAPK/ERK signalling pathway, we inhibited CRC cell metastasis. Transfected SW480 cells were incubated with PD98059, which is a MEK1/2 inhibitor, to decrease the phosphorylation of ERK. We found that PD98059 reversed the changes in the p-ERK, ZEB1, E-cadherin, and Snail proteins induced by PPP2R1B silencing in SW480 cells (Fig. 4A, B). In addition, PD98059 significantly reversed the cell invasion and migration regulated by PPP2R1B silencing. Specifically, compared with those in the PPP2R1B-siRNA1 group, the number of migrating and invading cells was markedly lower than that in the PPP2R1B siRNA1 plus PD98059 group (Fig. 4C, D). Moreover, compared with that in the PPP2R1B siRNA1 plus PD98059 group, the gap area in the PPP2R1B siRNA1 group was much larger (Fig. 4E, F). Taken together, these findings suggest that PPP2R1B inhibits CRC cell metastasis via the ERK/MAPK signalling pathway.

### PPP2R1B regulates Oxaliplatin sensitivity via the MAPK/ERK signalling pathway

**Fig. 5:**
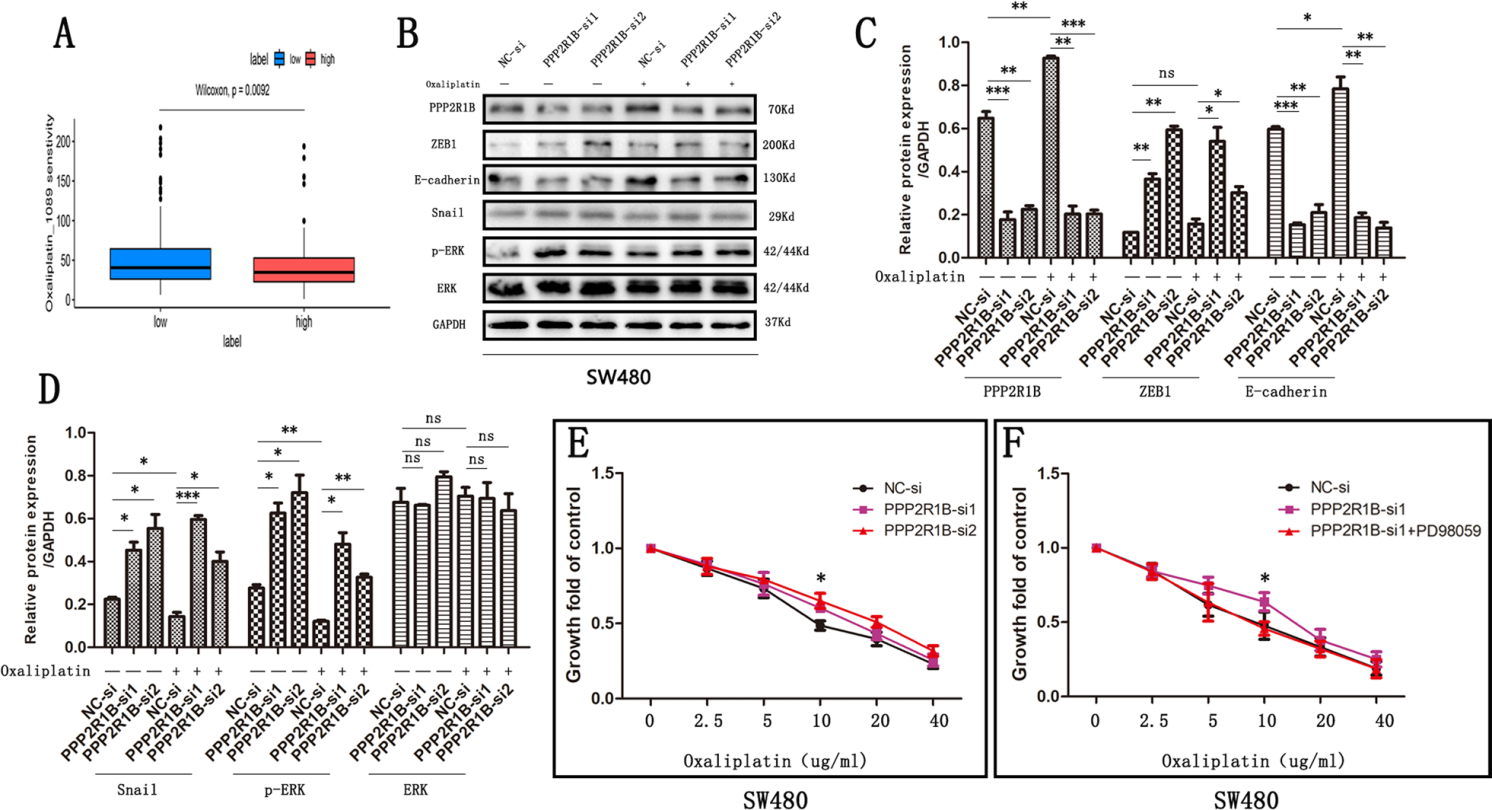
PPP2R1B regulate CRC cells Oxaliplatin chemosensitivity. **A** PPP2R1B mRNA level is associated with Oxaliplatin IC50 predicted by R package oncoPredict. **B** Protein of PPP2R1B, ZEB1, E-cadherin, Snail, p-ERK and ERK change in SW480 in PPP2R1B siRNA group and negative control group with or without Oxaliplatin treatment. **C**, **D** Quantitative analysis of the Western Blot was performed for SW480 cells using ImageJ software. **E** Chemosensitivity of SW480 treated with Oxaliplatin, in in PPP2R1B siRNA group and negative control group. **F** Chemosensitivity of SW480 treated with Oxaliplatin, in negative control group, PPP2R1B siRNA group and PPP2R1B siRNA plus PD98059 group. Bars indicate Mean ± S.E. *P < 0.05, **P < 0.01 and ***P < 0.001 compared with the control, n = 3

EMT not only regulates tumour cell metastasis but is also associated with drug resistance [9, 10]. Therefore, we utilized the R package oncoPredict (version = 0.2) to calculate 198 drug responses. Oxaliplatin and 5-fluorouracil (5-FU) are standard chemotherapy agents for CRC [19, 20]. We found that the PPP2R1B mRNA level was significantly related to the Oxaliplatin IC50 (Fig. 5A, p = 0.0092) but not to the 5-FU IC50 (p = 0.21). Therefore, we incubated NC-siRNA- or PPP2R1B siRNA-transfected cells with Oxaliplatin for 24 h. After treatment with 10 µM Oxaliplatin, the protein levels of PPP2R1B and E-cadherin were significantly elevated compared with those in the group without Oxaliplatin treatment. However, the protein levels of p-ERK and Snail decreased in the Oxaliplatin treatment group, while the levels of ZEB1 and ERK did not significantly change.

Furthermore, the CCK-8 assays showed that silencing PPP2R1B decreased the sensitivity of SW480 cells to Oxaliplatin (Fig. 5E). Moreover, PD98059 reversed the increase in Oxaliplatin chemosensitivity induced by PPP2R1B silencing (Fig. 5F).

### The prognostic value of the combination of PPP2R1B and p-ERK

**Fig. 6:**
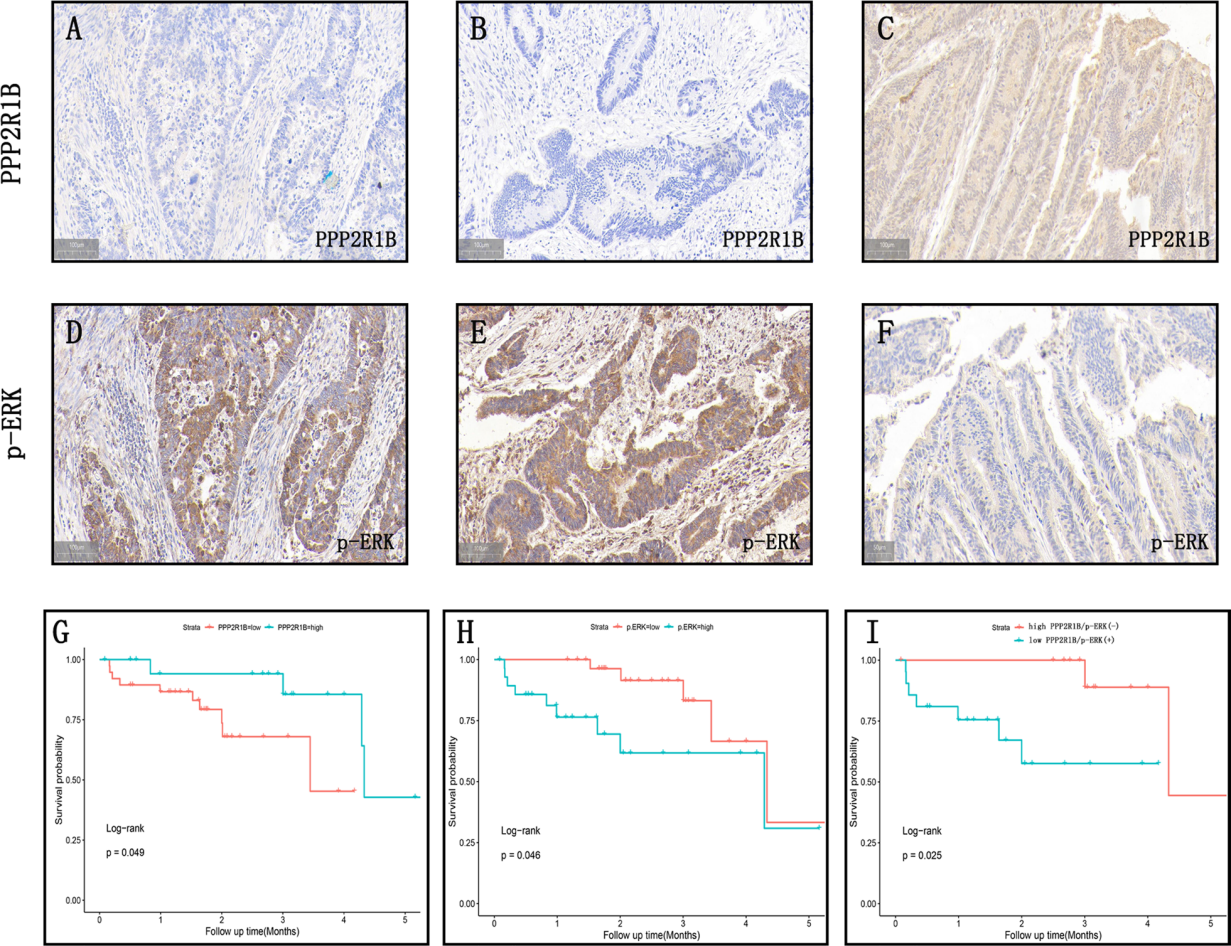
The negative relationship of PPP2R1B and p-ERK expression in human CRC samples was coordinately associated with the survival of CRC patients. **A**, **B** negative PPP2R1B and **D**, **E** positive p-ERK in two serial CRC samples. **C** positive PPP2R1B and **F** low p-ERK in another CRC samples. **G** Kaplan–Meier curves for patients with high versus low PPP2R1B expression in tumors. **H** Kaplan–Meier curves for patients with high versus low p-ERK expression in tumors. **I** Kaplan–Meier curves for patients with the combination of PPP2R1B and p-ERK

To evaluate the clinical relevance of PPP2R1B and p-ERK, we conducted IHC staining assays on these two proteins in 100 CRC tissues. The results showed that PPP2R1B and p-ERK staining patterns were the opposite in certain samples (Fig. 6A–F). Regression analysis revealed a significant negative correlation between the level of PPP2R1B and the level of p-ERK in CRC tissues (Table 2, r = − 0.32, p = 0.001). Based on the above finding that PPP2R1B suppresses CRC metastasis through the dephosphorylation of p-ERK, we investigated whether the combination of PPP2R1B and p-ERK could be used to predict CRC survival better than either protein alone. First, we found that the PPP2R1B-high group had better overall survival than the PPP2R1B-low group did (Fig. 6G, p = 0.049), and the p-ERK high-group had significantly shorter overall survival than the p-ERK-low group did (Fig. 6H, p = 0.046). Importantly, we also found that patients in the PPP2R1B-high/p-ERK-low subgroup had a better prognosis than did those in the PPP2R1B-low/p-ERK-high subgroup (Fig. 6I, p = 0.025). Taken together, these results indicate that the combination of PPP2R1B and p-ERK has potential value as a prognostic biomarker in CRC and that these proteins may be promising therapeutic targets in the clinic.

**Table 2:** The association of PPP2R1B and p-ERK in 0 CRC clinical samples

Parameter	PPP2R1B		r rank	P
	Low (n = 62)	High (n = 38)		
p-ERK			− 0.32	0.001
Low (n = 31)	13	18		
High (n = 69)	49	20		

### PPP2R1B silencing promotes CRC tumour metastasis in vivo

**Fig. 7:**
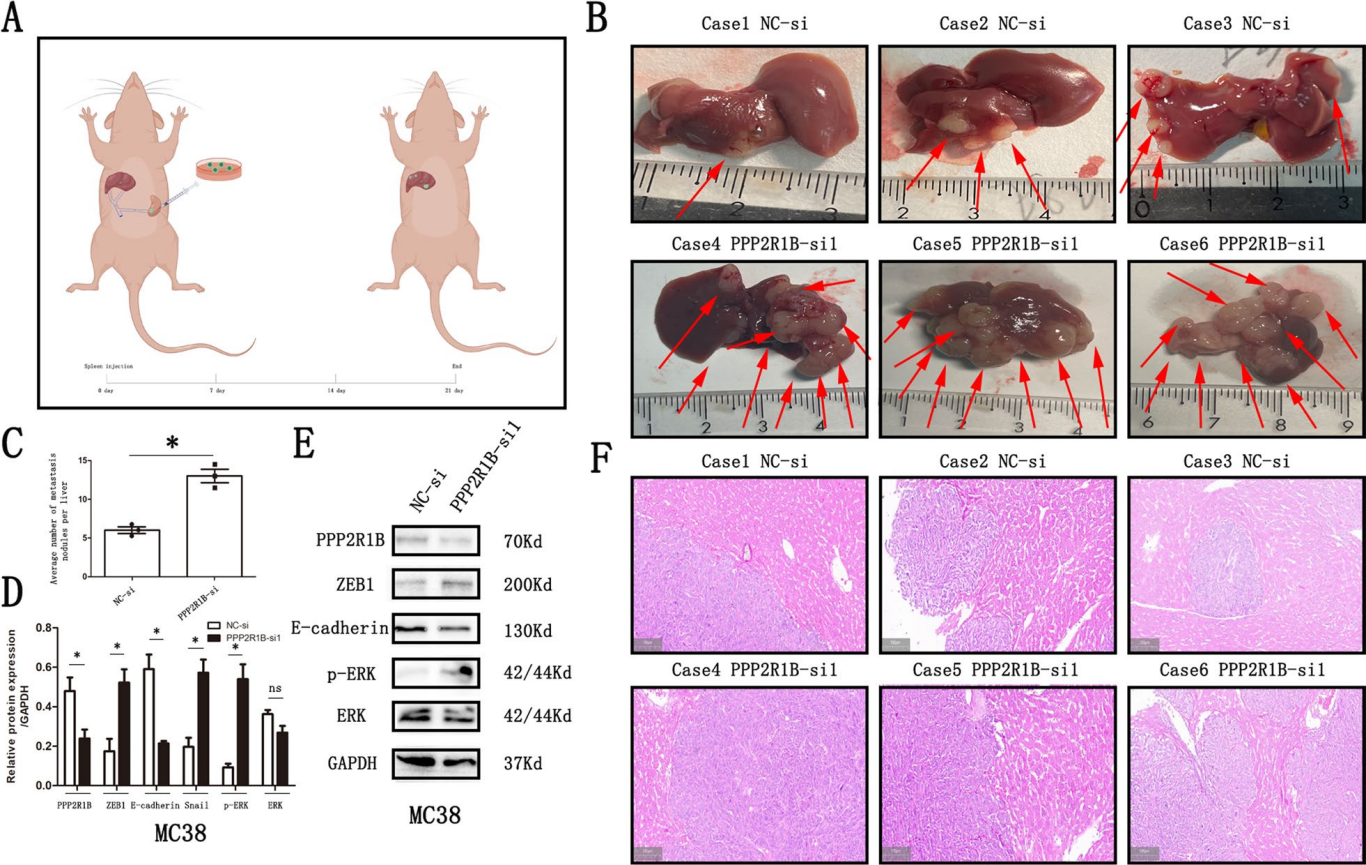
PPP2R1B interfering promoted CRC cells liver metastasis in vivo. **A** Cartoon diagram of mouse spleen infection liver metastasis model **B** C57BL/6 mice liver metastasis model in NC and PPP2R1B siRNA groups. **C** the numbers of liver metastasis in Negative Control siRNA and PPP2R1B siRNA groups. **D** Western Blot results of liver metastasis in two groups in vivo. **E** Quantitative analysis of the Western Blot was performed for MC38 cells using ImageJ software. **F** the HE staining result of liver metastasis tissue in vivo. Bars indicate Mean ± S.E. *P < 0.05, **P < 0.01 and ***P < 0.001 compared with the control, n = 3

MC38 cells with PPP2R1B knockdown and those expressing a negative control were injected into the spleens of nude mice. After a 21-day period, the livers were excised and imaged, and metastatic foci were counted (Fig. 7A–C). Western blotting further verified that PPP2R1B and E-cadherin expression levels were significantly deceased but ZEB1 and p-ERK expression levels were increased in PPP2R1B-si1 group compared with the NC-si group, while total ERK protein expression was not affected (Fig. 7D, E). HE staining revealed a larger area of liver metastasis in the PPP2R1B-si1 group than in the NC-si group (Fig. 7F). Thus, these results demonstrate that PPP2R1B plays a crucial role in modulating liver metastasis in vivo.

## Discussion

CRC is one of the most common malignant tumours, and metastasis and drug resistance are complex multistep processes that require the regulation of many cellular pathways. The prognosis of CRC with liver metastasis or drug resistance is poor [3, 4]. In this study, we demonstrated the important role of PPP2R1B as a suppressor gene for CRC liver metastasis via analysis of a high-throughput sequencing dataset and explored its prognostic value in CRC cohort. Overall, we elucidated the potential molecular mechanisms by which PPP2R1B regulates CRC metastasis and Oxaliplatin chemosensitivity. Specifically, we found that PPP2R1B binds to p-ERK and induces the dephosphorylation of p-ERK and subsequently inhibits the MAPK-ERK signalling pathway both in vitro and in vivo, which suggests that PPP2R1B may be a potential therapeutic target in CRC.

PPP2R1B is located at chromosome 11q23.1 and is abnormally expressed in colon, breast and lung cancers [21, 22]. Previous studies have reported that PPP2R1B is a tumour suppressor that plays a role in tumorigenesis [23, 24]. Moreover, the inhibition of miR-587 or restoration of PPP2R1B expression may have significant therapeutic potential for overcoming drug resistance in CRC patients, and the combined use of an AKT inhibitor with 5-FU may increase the efficacy of CRC treatment [25]. In tongue squamous cell carcinoma (TSCC), exosomal miR-200c may be an effective strategy for suppressing chemoresistance to docetaxel, as it inhibits TUBB3 and PPP2R1B [8]. However, the biological functions and molecular mechanisms of PPP2R1B in tumorigenesis are unclear. First, the genes related to liver metastasis and survival-related in the public dataset were overlapped, and PPP2R1B was identified as the only common gene. These findings indicate that PPP2R1B plays a significant inhibitory role in CRC liver metastasis. Subsequently, via IHC staining, we confirmed that PPP2R1B expression was obviously lower in CRC tissues and even lower in CRC liver metastases than in corresponding adjacent noncancerous tissues. Our findings are in line with the bioinformatics analysis results. Moreover, a series of functional assays showed that PPP2R1B could inhibit CRC metastasis in vitro and in vivo. Taken together, our findings revealed that PPP2R1B is a key suppressor gene in CRC metastasis.

However, to date, studies on the biological function and molecular mechanism of PPP2R1B in CRC are rare. Regression analysis was used to analyse the relationship between pathways identified by GSVA based on the C6: oncogenic signature gene sets from GSEA (https://www.gsea-msigdb.org/gsea/msigdb/index.jsp) and PPP2R1B. Regression analysis combined with LC–MS/MS revealed that PPP2R1B negatively regulates the MEK signalling pathway. p-ERK is a core gene in the MEK signalling pathway. Additionally, protein phosphatase 2A (PP2A), a protein complex containing PPP2R1B, is reported to regulate several significant signalling pathways, such as the MAPK/ERK and PI3K/AKT pathways [26, 27]. However, there is no evidence demonstrating the direct interaction between PPP2R1B and p-ERK. Our study verified for the first time that PPP2R1B, a phosphatase, binds to the p-ERK protein, which was verified by coimmunoprecipitation analysis, and subsequently induces the dephosphorylation of p-ERK and the inhibition of the MAPK/ERK signalling pathway, which was verified by Western blot analysis. Finally, we verified the relationship between PPP2R1B and p-ERK protein expression in CRC tissues by IHC staining. These results suggested that p-ERK may be a substrate of PPP2R1B.

It is now well recognized that EMT promotes the malignant progression of CRC and promotes cell invasion, migration, and drug resistance [28, 29]. EMT is typically characterized by a decrease in the epithelial markers E-cadherin and ZO1 and upregulation of the EMT-related transcription factors ZEB1, Snail, Slug and so on [30]. As transcription factors of E-cadherin, ZEB1 and slug bind to the E-cadherin promoter to suppress its transcription and trigger tumour cell dedifferentiation and spreading, which is the key step of the EMT process [31, 32]. Moreover, ZEB1 and Snail are reportedly regulated by the MAPK-ERK signalling pathway in cancers [33, 34]. Western blot analysis also showed that PPP2R1B silencing stabilized p-ERK and altered the expression of ZEB1, Snail and E-cadherin. A MAPK/ERK signalling pathway inhibitor (PD98059) disrupted the ability of PPP2R1B to regulate EMT. Taken together, these findings show for the first time that PPP2R1B silencing promotes MAPK/ERK signalling and EMT via ZEB1 and Snail.

Xelox is widely used as a first-line chemotherapeutic regimen for primary CRC. However, the response is unsatisfactory due to the lack of effective predictive markers of sensitivity to treatment [35, 36]. It has been shown that EMT plays an important role in the progression of drug sensitivity [37]. We verified that PPP2R1B could regulate EMT, so we further investigated the role of PPP2R1B in increasing Oxaliplatin sensitivity. The IC50 of Oxaliplatin was predicted by the R package OncoPredict. A bar chart showed that PPP2R1B could identify patients who are more likely to be sensitive to Oxaliplatin. In Oxaliplatin-treated SW480 CRC cells, PPP2R1B was significantly elevated. Furthermore, coimmunoprecipitation revealed that Oxaliplatin increases the binding capacity of PPP2R1B to the p-ERK complex and subsequently increases or decreases the expression of E-cadherin and Snail, respectively. Finally, CCK-8 assays were performed, and silencing PPP2R1B decreased the sensitivity of CRC cells to Oxaliplatin. In addition, PD98059 significantly reversed the decrease in Oxaliplatin sensitivity induced by silencing PPP2R1B. At least one study has also demonstrated that the MAPK/ERK pathway regulates drug sensitivity [38]. Taken together, these findings suggest that PPP2R1B negatively regulates p-ERK protein expression and increases Oxaliplatin sensitivity.

## Conclusion

**Fig. 8:**
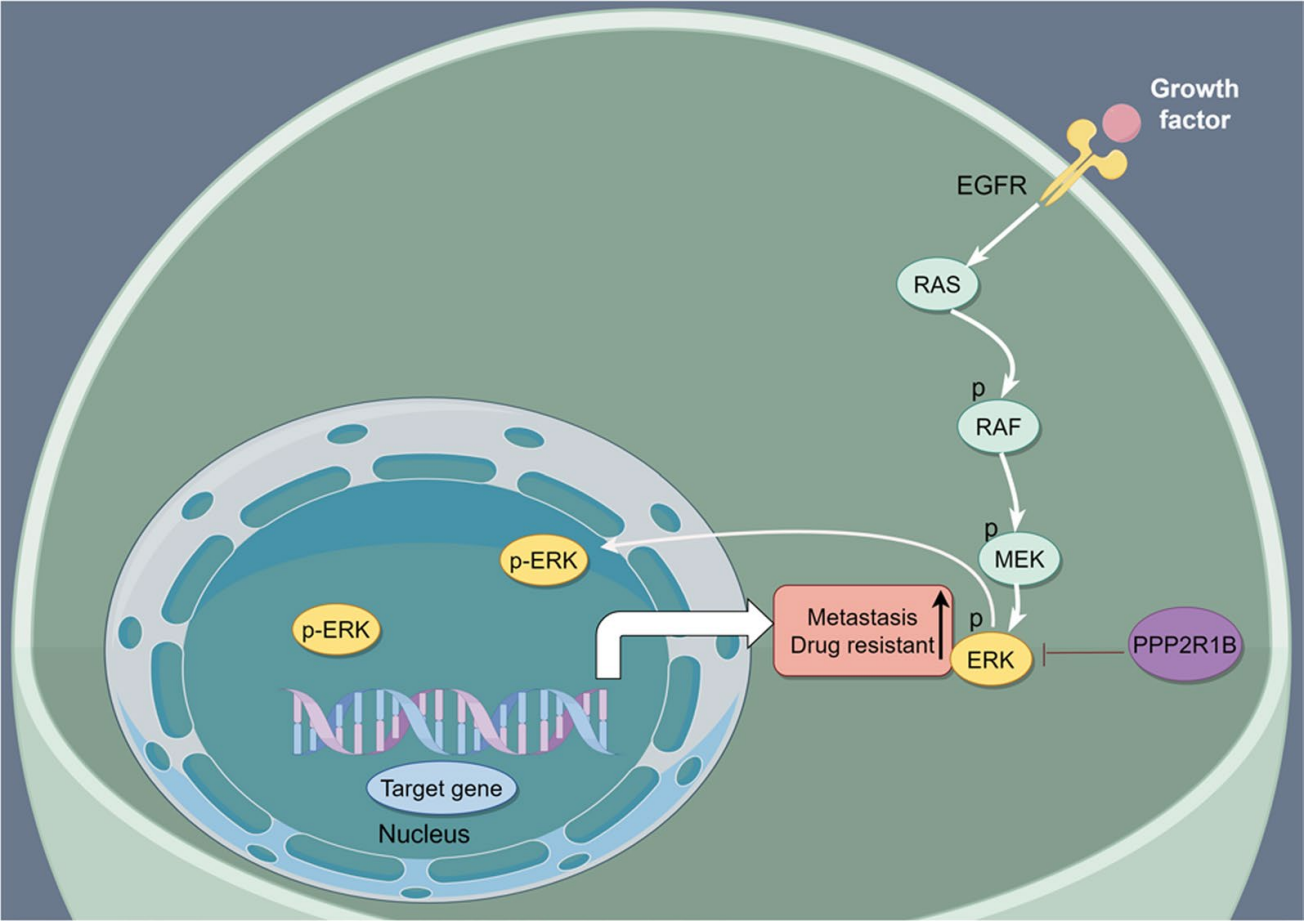
The proposed mechanism by which PPP2R1B regulates CRC cells metastasis promotes CRC cells Oxaliplatin sensitivity by MAPK/ERK signaling pathway. PPP2R1B binds to p-ERK and induce dephosphorylation of p-ERK

We provided compelling evidence that PPP2R1B is downregulated in CRC patients and predicts a good prognosis and good clinical outcomes. We also elucidated the critical role of PPP2R1B in inhibiting CRC metastasis and increasing Oxaliplatin sensitivity: it acts by regulating the MAPK/ERK signalling pathway. Mechanistically, PPP2R1B binds to p-ERK and induces the dephosphorylation of p-ERK (see Fig. 8). Furthermore, PPP2R1B may be a potential candidate for therapeutic application in CRC.

### Supplementary Information

**Additional file 1. Table S1.** The result of Shotgun LC-MS/MS.

## Data Availability

Any reasonable requests for access to available data underlying the results reported in this article will be considered. Such proposals should be submitted to the corresponding author.

## Abbreviations

CRC: Colorectal cancer
PPP2R1B: Protein Phosphatase 2 Scaffold Subunit Abeta
DEGs: Differentially expressed genes
TCGA: The Cancer Genome Atlas
GEO: Gene Expression Omnibus data base
GSVA: Gene set variation method
LC–MS/MS: Liquid Chromatography Tandem-mass Spectrometry
IHC: Immunohistochemistry
qRT-PCR: Quantitative real‑time PCR
EMT: Epithelial‑mesenchymal transition
ZEB1: Zinc Finger E-Box Binding Homeobox 1
MAPK: Mitogen-Activated Protein Kinase 1
5-FU: 5-Fluorouracil
